# A Complete Sequence and Transcriptomic Analyses of Date Palm (*Phoenix dactylifera L*.) Mitochondrial Genome

**DOI:** 10.1371/journal.pone.0037164

**Published:** 2012-05-24

**Authors:** Yongjun Fang, Hao Wu, Tongwu Zhang, Meng Yang, Yuxin Yin, Linlin Pan, Xiaoguang Yu, Xiaowei Zhang, Songnian Hu, Ibrahim S. Al-Mssallem, Jun Yu

**Affiliations:** 1 Joint Center for Genomics Research (JCGR), King Abdulaziz City for Science and Technology (KACST) and Chinese Academy of Sciences (CAS), Riyadh, Kingdom of Saudi Arabia; 2 James D. Watson Institute of Genome Sciences, Zhejiang University, Hangzhou, China; 3 CAS Key Laboratory of Genome Sciences and Information, Beijing Institute of Genomics (BIG), Chinese Academy of Sciences (CAS), Beijing, China; 4 Department of Biotechnology, College of Agriculture and Food Sciences, King Faisal University, Hofuf, Kingdom of Saudi Arabia; University of Veterinary Medicine Hanover, Germany

## Abstract

Based on next-generation sequencing data, we assembled the mitochondrial (mt) genome of date palm (*Phoenix dactylifera L*.) into a circular molecule of 715,001 bp in length. The mt genome of *P. dactylifera* encodes 38 proteins, 30 tRNAs, and 3 ribosomal RNAs, which constitute a gene content of 6.5% (46,770 bp) over the full length. The rest, 93.5% of the genome sequence, is comprised of cp (chloroplast)-derived (10.3% with respect to the whole genome length) and non-coding sequences. In the non-coding regions, there are 0.33% tandem and 2.3% long repeats. Our transcriptomic data from eight tissues (root, seed, bud, fruit, green leaf, yellow leaf, female flower, and male flower) showed higher gene expression levels in male flower, root, bud, and female flower, as compared to four other tissues. We identified 120 potential SNPs among three date palm cultivars (Khalas, Fahal, and Sukry), and successfully found seven SNPs in the coding sequences. A phylogenetic analysis, based on 22 conserved genes of 15 representative plant mitochondria, showed that *P. dactylifera* positions at the root of all sequenced monocot mt genomes. In addition, consistent with previous discoveries, there are three co-transcribed gene clusters–*18S-5S rRNA*, *rps3-rpl16* and *nad3-rps12*–in *P. dactylifera,* which are highly conserved among all known mitochondrial genomes of angiosperms.

## Introduction

The widely-accepted hypothesis about the origin of the mitochondrion assumes that it descended from an endosymbiontic event involving an α-proteobacterium-like organism and the common ancestor of eukaryotes [1]. Evolving from algae to land plants, including bryophytes and angiosperms, plant mitochondrial (mt) genomes have increased their sizes, especially in the non-coding region. Among land plants, bryophytes, *i*.*e*., liverworts, mosses, and hornworts, represent the basal forms. They have similar gene order, genome size, and a fraction of non-coding sequences [2], [3]. As evolution continues, land plants gained new mechanisms to facilitate frequent gene exchange between mitochondrial and chloroplast genomes as well as between mitochondrial and nuclear genomes [4], [5]. For instance, mitochondrial genomes of angiosperms have long been known for their slow evolutionary rate [6], existence of subgenomic circles in addition to a master genomic circle [7], extraordinarily large and highly variable genome sizes [8], trans-splicing of group II introns [9], high density of RNA editing [10], [11], divergent non-coding sequences [12], and frequent gene transfer [13]. The inter-genomic gene transfer, together with the continuing increase of non-coding DNA sequences, leads to a broad size range in angiosperm mt genomes, which as of today is from ∼200 to 2400 kb based on the known mt sequences and experimental estimations [8], [14]; up to date (July, 2011), there have been more than 40 plant mt genomes sequenced, including 22 angiosperm mt genomes (http://www.ncbi.nlm.nih.gov/genomes/GenomesGroup.cgi?taxid=33090&opt=organelle).

*Phoenix dactylifera L.,* also known as date palm, is economically the most important plant in the Middle East and North Africa [15], and it is estimated to have more than 450 cultivars or varieties in the Kingdom of Saudi Arabia and nearly 2,000 varieties around the world [16]. Therefore, sequencing its mitochondrial genome, together with its nuclear [17] and chloroplast genomes [18], is of essence in improving its agricultural, horticultural, and nutritional values. In this study, combining data from two next-generation sequence platforms, pyrosequencing (Roche GS FLX) and ligation-based sequencing (Life Technologies SOLiD), we assembled *P. dactylifera* mt-genome (cultivar Khalas, Al-Hasa Oasis, Saudi Arabia) –the first from the *Arecaceae* family. In addition, analysis of the mt genome sequence and transcriptomic data are of importance in revealing mechanisms underlying mitochondrial genome evolution and the unique evolutionary status of *P. dactylifera* among angiosperms. Furthermore, based on the data from three commonly-grown cultivars, we also investigated RNA editing sites and SNPs within the species.

## Results and Discussions

### General Features of *P. dactylifera* L. mt Genome

**Figure 1:**
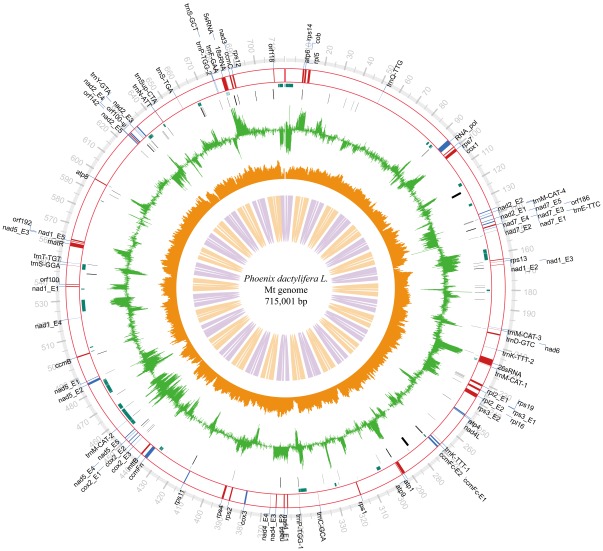
A circular display of *P. dactylifera* mitochondrial genome. We display (starting from outside to inside): physical map scaled in kb, coding sequences transcribed in the clockwise (red) and counterclockwise directions (blue), chloroplast-derived regions (green boxes), sequence repeats (black), histogram of transcriptome data (green bar, standing for average RPKM value per 200 bp, transformed using natural logs and ranging from 0 to 10), GC content variations (brown bar in a 500 bp sliding window and 500 bp increments), and SOLiD mate-pair (MP) read validation (sliding window 2 kb, MP insertion size 5–6 kb, Step size 15 kb). This figure was generated by using the Circos program [68]. Ψ indicates pseudogene.

**Figure 2:**
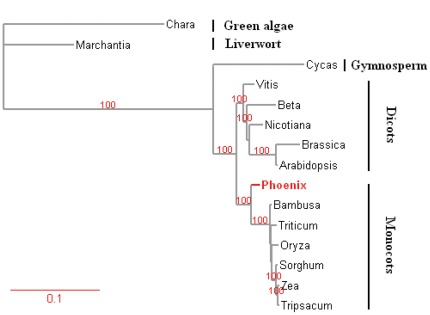
Phylogeny inferred from 22 genes common to 15 plant mt genomes. We constructed an ML tree using PHYML (version 3.0) [67] (*Chara vulgaris* as outgroup, see Materials and Methods for details). Nodes receive over 90% bootstrap replicates are indicated. *P. dactylifera* mt genome rooted at the basal position of monocots (red).

We assembled the *P. dactylifera* master mt chromosome into a 715,001 bp circular molecule (Figure 1; the assembling details are described in Materials and Methods) with an average GC content of 45.1%; it is now the fourth largest mt genome sequenced after those of *Cucumis sativus* (1,555,935 bp) [19], *Cucurbita pepo* (982,833 bp), and *Vitis vinifera* (773,279 bp). Its protein coding sequence is composed of only 6.5% of the genome (46,770 bp) and this gene content is similar to other published angiosperm mt-genomes (Table S1 and Table S2). The rest, also the majority (93.5%) of the genome, is composed of non-coding (the cp-derived regions are also considered as non-coding in this regard), which harbors 0.33% tandem and 2.3% long repeats (the repeat lengths are greater than 50 bp). RNA genes and intron sequences are 1.1% and 4.3% of the mt genome, respectively. This mt genome also contains the second highest proportion (10.3%) of cp-derived sequences among the sequenced mt genomes to date, of which several intact genes, such as *petA, petG, petL, psaJ, psbT, rpl20, rpl33,* and *rps8* are identified. Since the age of the cp-derived sequences or time when the sequences inserted into mt genomes varies greatly [20], we are unable to prove whether these genes are actually transcribed or active since we extracted the total RNA (contains both nuclear and organellar transcripts) from each tissue for constructing transcriptomic libraries among which the expression data of the cp-derived sequences and authentic cp sequences are impossible to separate (see Materials and Methods for more details). Most of *P. dactylifera* mt genome is rather diverged from other angiosperms. For example, only ∼21% of *P. dactylifera* mt genome sequence is shared (over 70% identity) by *Vitis*, *Oryza* and *Bambusa*, and even less by *Zea* (∼15%) and *Arabidopsis* (∼11%). In addition, consistent with results from previous studies, we observed that three co-transcribed gene clusters, *18S-5S rRNA*, *rps3-rpl16*, and *nad3-rps12*, are conserved in other angiosperm mt genomes [21]. We summarized general mt genome features including size variations, AT content, and intron types of 15 non-redundant sequenced plant mt genomes (including 12 higher plants and three lower plants) in Table 1. Our phylogenetic analysis based on 22 concatenated conserved genes among 15 selected mt genomes (Figure 2) revealed that *P. dactylifera* appears to be the more basal among monocots.

**Table 1:** Comparative analysis of genomic features among 15 mt genomes.

	Size(bp)	AT(%)	Gene number (Total/Protein/tRNA/rRNA)	Coding(%)	Repeats(%)^b^	Cp(%)	Group I introns	Group II introns (Cis/Trans-spliced)	RNA editing sites
*Chara* a	67,737	59.1	76/46/27/3	90.7	1.5	−	14	13/0	−
*Marchantia* a	186,609	57.6	110/76/29/3	20.3	7.8	−	7	25/0	−
*Cycas* a	414,903	53.1	70/39/26/3	10.1	21	4.4	0	20/5	1084
*Beta* a	368,801	56.1	171/140/26/5	10.3	13.4	2.1	0	14/6	370
*Brassica* a	221,853	54.8	100/79/17/3	17.3	5.2	3.6	0	18/5	427
*Arabidopsis* a	366,924	55.2	131/117/21/3	10.6	10.6	1.1	0	18/5	441
*Nicotiana* a	430,597	55.0	183/156/23/4	9.9	11.7	2.5	0	17/6	−
*Vitis*	773,279	55.9	161/74/31/3	5.0	2.9	8.8	0	−/−	−
*Phoenix*	715,001	54.8	90/43/23/3	6.5	2.3	10.3	0	20/4	592
*Bambusa*	509,941	56.1	61/35/21/5	6.3	5.5	−	0	−/−	−
*Triticum* a	452,528	55.7	78/39/34/9	8.6	15.9	3.0	0	17/6	−
*Oryza* a	490,520	56.2	81/53/22/3	11.1	30.4	6.3	0	17/6	446
*Sorghum*	468,628	56.3	54/32/18/3	6.7	16.2	−	0	−/−	−
*Tripsacum*	704,100	56.1	55/33/18/3	5.0	36.4	−	0	−/−	−
*Zea* a	569,630	56.1	213/163/33/4	6.2	19.1	4.4	0	15/7	−

We summarized several genomic features from 15 representative mt genomes, including AT content of the mt genomes, the percentage of gene-coding sequences, and the percentage of chloroplast-derived sequences in mt genome sequences. We only used the genus names for the reference genomes.

a Information about these mt genomes are from reference [69] and information about other plant mt genomes are either from original publications or NCBI databases (see Table S1).

b To be consistent, repetitive sequence contents in the 15 plant mt genomes are all computed by using REPuter (length >50 bp; mismatch ≤3).

### Protein Coding, rRNA, and tRNA Genes

The *P. dactylifera* mt genome contains at least 38 protein-coding genes and five complete ORFs, most of these genes encode proteins of the electron transport chain, such as nine subunits of nicotinamide adenine dinucleotide dehydrogenase (complex I), apocytochrome b (complex III), three subunits of cytochrome c oxidase (complex IV), five subunits of ATP synthase F1 (complex V) and four cytoplasmic membrane proteins required for cytochrome c maturation (Table 2).

**Table 2:** The gene content of *P. dactylifera* mt genome.

Genes of Mitochondrial Origin	
Complex I	*nad1, nad2, nad3, nad4, nad4L, nad5, nad6, nad7,* and *nad9*
Complex III	*cob*
Complex IV	*cox1,cox2,* and *cox3*
Complex V	*atp1, atp4, atp6, atp8,* and *atp9*
Cytochrome c biogenesis	*ccmB, ccmC, ccmFc,* and *ccmFn*
Ribosome large subunit	*rpl2, rpl5,* and *rpl16*
Ribosome small subunit	*rps1, rps2, rps3, rps4, rps7, rps11, rps12, rps13, rps14,* and *rps19*
Intron maturase	*matR*
SecY-independent transporter	*mttB*
rRNA genes	*5sRNA,18sRNA,* and *26sRNA*
tRNA genes	*trnC-GCA, trnD-GTC, trnE-TTC, trnF-GAA, trnK-TTT(×2), trnM-CAT(×4), trnN-ATT, trnP-TGG(×2), trnQ-TTG, trnS-GCT, trnS-GGA, trnS-TGA, trnSup-CTA, trnT-TGT,* and *trnY-GTA*
Pseudogenes	*orf100-ψ*
Hypothetical genes	5 ORFs
**Genes of Chloroplast Origin**	
Genes with intact ORFs^a^	*accD, atpI, cemA, infA, matK, ndhI, ndhJ, petA, petB, petG, petL, psaB, psbA, psbE, psbH, psbJ, psbL, psbN, psbZ, rpl14, rpl33, rpl36, rpoA, rps14, rps2, rps4,* and *ycf4*
Pseudogenes	*psbT* and *rps18*
tRNA genes	*trnC-GCA, trnF-GAA, trnG-GCC, trnH-GTG, trnM-CAT, trnN-GTT, trnP-GGG,trnS-TGA,* and *trnW-CCA(×2)*
**Genes of Nuclear Origin**	
RNA polymerase	RNA_pol

a Genes with intact ORFs in cp-derived regions are identified based on >95% identity and >95% length coverage to the known cp genes.

We compared these protein coding genes to 11 other sequenced angiosperm mt genomes (Table S2). *First*, *P. dactylifera* mt genome does not have the genes encoding respiratory chain complex II, such as *sdh3* and *sdh4,* which are only found in two dicots, *Nicotiana tabacum* and *V. vinifera*. *Second*, our assembly is similar to *V. vinifera*, and both contain one copy of RNA polymerase gene harboring a conserved domain characteristic of pfam00940 superfamily of polymerases [22]. *Third*, *rps14* present in *Brassica napus* and *V. vinifera* is also found here, whereas *rps11*, another ribosomal protein gene, is exclusively detected in our assembly. Both genes have full open reading frame (ORF) and are likely functional in date palm, though in many other known angiosperm mt genomes they are either pesudogenes or transferred into nuclear genomes [23], [24]. *Fourth*, the recently identified *rpl10* gene, being identified as *orf-bryo1* in vascular plants and charophycean green algae [25] and *orf168*-related sequences in bryophytes and angiosperms [26], seems to be interrupted in our assembly and possibly because of a frame shift event. *Fifth*, we found several pseudogenes in our assembly, which appear intact in other mt genomes, such as *orf99-b* (as orf100-ψ in our gene list) in *Zea mays* and cp-derived gene *psbT* in *V. vinifera*. In addition, some of the universally expressed ribosomal genes, including three rRNA genes (5S, 18S, and 26S ribosomal RNA genes), are also unambiguously identified [27]; 5S and 18S rRNA genes are also closely related and distant from 26S rRNA gene in date palm mt genome.

**Figure 3:**
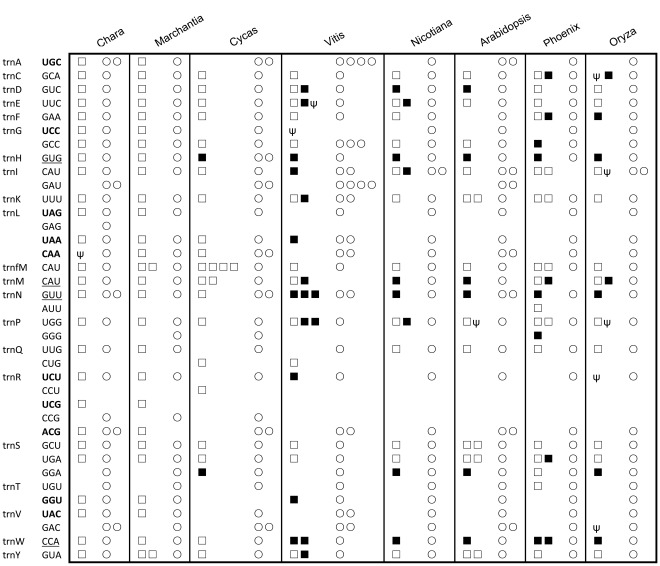
The distribution of tRNAs in vascular and angiosperm plant mitochondrial and chloroplast genomes. Native tRNA genes in mitochondrial and chloroplast genomes are shown in open square and circles, respectively. Solid squares indicate cp-derived tRNAs found in mt genome and ψ stands for pseudogene. There are ten tRNAs (their anticodons are highlighted in bold) that are gradually lost in genome evolution and four tRNAs (their anticodons are underlined) that are gradually replaced by their cp-derived counterparts. These eight mt genomes are listed according to their relative phylogenetic positions in Figure 2.

A genome-wide screening yielded 30 full-length tRNA sequences (Table S3) in our assembly; among them, 12 seem to be cp-derived, which exhibit higher sequence identity (>98%) to their chloroplast counterparts than their mitochondrial counterparts [18], and three predicted tRNAs seem to have introns. Among these 30 tRNA genes five amino acids (A, L, R, T, and V) are not encoded, although tRNAs for 20 amino acids are necessary for protein synthesis in mitochondria. In addition, having compared the date palm tRNA gene content to those of seven other plants mt and cp genomes (Figure 3), we conclude that there are 10 tRNA genes, among which nine encoding tRNAs for the five amino acids, are actually lost after the divergence of liverworts from seed plants. These results suggest that the missing tRNAs are supplied by either the chloroplast or nuclear genomes. In addition, we found that four mt tRNA genes of higher plants are gradually lost and replaced by cp-derived tRNA [28]. The reason why mt tRNA genes are replaced by both cp-derived and nuclear counterparts remains an open question. There is also another possibility–all mt tRNA genes may eventually be replaced and what we observed here is only an intermediate and dynamic process.

### Plastid DNA Insertions

Chloroplast and mitochondrial genomes are known to share sequences due to frequent gene transfer events [5], [29], [30]. Frequent DNA transfer from cpDNA to mtDNA occur as far back as the common ancestor of the extant gymnosperms and angiosperms, about 300 MYA (million-years-ago) [20]. Our assembly contains more than 100 fragments of chloroplast origin (over 80% identity), ranging from 50 to 6,521 bp in length (Table S4). The total fraction of chloroplast DNA sequences present in *P. dactylifera* mt genome is 73,691 bp, corresponding to 10.3% of the whole mt genome, and 46.5% of *P. dactylifera* cp-genome. The proportion of cp-derived sequences in our mt genome assembly is comparable to the two large sequenced plant mt genomes, *V. vinifera* (8.8%) and *Cucurbita pepo* (11.6%) [31], but larger than those of the other known plant mt genomes (Table 1). These results suggest that chloroplast DNA sequence insertion is an important mechanism for plant mitochondrial genome size expansion and sequence diversity.

Most of chloroplast sequence insertions in *P. dactylifera* mt genome are unique, as evident from the observation that only nine out of 44 insertions (over 200 bp) have full-length homologous sequences shared by other known mt genomes (>90% length coverage, >70% identity) (Table 3). Among the nine cp-derived homologous sequences, six and five of them are also found in *Vitis* and *Bambusa*, respectively, whereas none is found in *A. thaliana*. These nine insertions tend to have higher GC content, resembling that of mt genomes as compared to the unique and possibly new insertions (Figure S1), which suggests that these cp-derived sequences did, in some extent, gradually increase their GC content to become similar to their host mtDNAs.

**Table 3:** The distribution of nine *P.dactylifera* chloroplast-derived mt regions in five known plant mt-genomes.

Position	Length	Identity^a^	GC^b^	*Arabidopsis*	*Vitis*	*Bambusa*	*Oryza*	*Zea*
130051–131335	1285	90	0.4084	−	+	−	−	−
87871–88837	967	88	0.4224	−	−	+	+	+
328935–329833	899	90	0.4405	−	+	+	+	+
179701–180523	823	91	0.4702	−	−	+	−	−
535882–536375	494	94	0.4231	−	+	−	−	−
271397–271847	451	87	0.3792	−	+	+	−	−
483235–483594	360	89	0.4389	−	+	−	−	−
586885–587154	270	95	0.4870	−	−	+	+	+
500598–500864	267	88	0.4607	–	+	−	−	−

We selected homologous sequences with identity >70% and length coverage >90% for the comparative analysis. The results for two dicots (*Arabidopsis* and *Vitis*) and three monocots (*Bambusa, Oryza, and Zea*) are listed here. The presence (+) and absence (−) of the corresponding cp regions are indicated based on identity and length coverage. Only the genus names are used for the reference mt genomes.

a The sequence identity between the cp sequence insertions in *P. dactylifera* mt genome and their cp homologs.

b The GC content of the cp-derived sequences in *P. dactylifera* mt genome.

### Introns and RNA Editing

We identified 23 group II introns in 10 protein-coding genes, including four trans-spliced introns of *nad1* and *nad5*, and 20 cis-spliced introns in *ccmFc*, *cox2*, *nad1*, *nad2*, *nad4*, *nad5*, *nad7*, *rpl2*, and *rps3*. No group I intron was discovered in our assembly. In general, the functional mitochondrial rRNA and tRNA genes of the sequenced angiosperm mt genomes do not possess introns, but we found three intron-containing tRNA genes in our assembly: *trnK-TTT*, *trnN-ATT* and *trnSup-CTA*, and we have yet to validate if they are functional or not.

**Figure 4:**
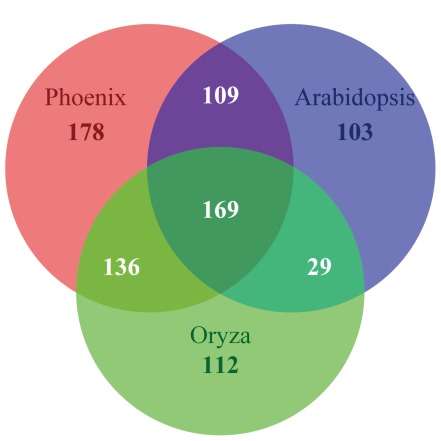
Venn diagram of shared RNA editing sites among three plant mt genomes.

Mitochondrial RNA editing is essential for functional protein synthesis since nearly all plant mt mRNAs are edited [32], [33], [34] and it modifies amino acids and generates new start or stop codons [35], [36], [37], [38], [39], and it has been documented in most plants except algae and mosses. It suggests that this cellular process is ancient arisen in early land plants after they split from Bryophyta [10]. We predicted nearly 600 putative RNA editing sites (Table S5) using PREP-Mt [40]–an effective tool identifying C-U editing sites. We found that the *nad4* gene contains the most edited sites (59). In addition, our comparative analysis revealed that 305 (51.5%) and 278 (47.0%) C-U editing sites in date palm are shared by *O. sativa* and *A. thaliana*, respectively (Figure 4). Experimental examination confirmed 40 of 41 predicted C-U editing sites in five randomly chosen genes (*atp1*, *atp4*, *atp9*, *rpl116* and *rps19*) using cDNA sequences (Table S6) and additional nine sites not detected by PREP-Mt were identified. We also compared their tissue disparity between mRNA transcripts extracted from yellow and green leaves, but no obvious tissue-specific RNA editing patterns are yet identified among these five genes, although reports in the literature indicated that the extent of *atp6* editing is significantly different among tissue types [41]. Therefore we assume the tissue-specific RNA editing patterns may be only detectable in certain genes, cell types, and developmental stages [42].

### SNP Analysis

Plant cells usually possess hundreds to thousands mitochondria or copies of mt genomes that can be regarded as a population when genetic heterogeneity is to be investigated. High throughput next-generation sequence technologies provide us the opportunity to survey single nucleotide polymorphisms in the same or different species (subspecies or cultivars) by mapping reads to a reference sequence and to each cultivar. The polymorphisms within the same cultivar genome (intra-varietal SNPs) and among different cultivars genomes (inter-varietal SNPs), discovered by high-coverage of reads, can also be separated into major and minor genotypes based on simple read counts. Here, we use three runs of SOLiD fragment data from each of the three cultivars (Khalas, Fahal, and Sukry) sequenced in our study for intra-varietal (Table 4) and inter-varietal SNP identification (Table 5). We identified 651, 703, and 731 intra-varietal SNPs in cultivar Khalas, Fahal, and Sukry, respectively, estimated to have a polymorphism rate of one in 1,000 bp, which is about two times higher than that of date palm chloroplast [18] but is only about one tenth of rice mt genome [43]. We should be cautious here since different SNP analysis methods are applied because of the distinct sequencing strategies used in sequencing these mt or cp genomes. The rates of each variation type among these intra-varietal SNPs of the three cultivars are very similar except the types (such as A to T or G to C and vice versa) that do not change GC contents are less represented. These SNPs can also be separated into transition and transversion types, and as a result, there are 297, 325, 287 transitions and 354, 378, 347 transversions for Khalas, Fahal, and Sukry, respectively. The rate of transversions is slightly higher than that of transitions, though in chloroplast transversion (52) is 2× that of transition (26) [18].

**Table 4:** Intra-varietal SNPs among the three cultivars.

SNP^a^	Khalas^b^	Fahal	Sukry
**Transition**			
A/G	72	75	68
G/A	83	99	85
T/C	66	66	64
C/T	76	85	70
total	297	325	287
**Transversion**			
A/C	78	88	74
A/T	28	32	33
C/A	60	60	60
C/G	11	14	10
G/C	10	12	12
G/T	65	63	63
T/A	31	34	33
T/G	71	75	62
Total	354	378	347

a Major and minor genotypes are separated with oblique lines (/).

b Numbers of sites are calculated for each cultivar.

**Table 5:** Inter-varietal SNPs.

	Coding^a^	Non-coding^a^	Total
Khalas vs. Fahal	2	79	81
Khalas vs. Sukry	6	91	97
Fahal vs. Sukry	6	50	56
Khalas vs. Fahal vs. Sukry	7	113	120

a “Coding" and “Non-coding" indicate numbers of inter-varietal SNPs found among the groups.

All together, there are 120 candidate SNP sites identified among the three cultivars (Table 5), with an inter-varietal polymorphism rate of 0.017%, similar to that of subspecific (between subspecies) polymorphisms between rice cultivar *93-11* and *PA64S,* ∼0.02% [43]. The inter-varietal SNPs are predominantly found in non-coding regions, only seven SNPs were found in coding sequences (all are located in 26S and 18S rRNA genes; Table S7): two between Khalas and Fahal, six between Khalas and Sukry, and six between Fahal and Sukry (Table 5). As to the remaining 113 inter-varietal SNPs residing in non-coding regions (Table S8), 79 of them are between Khalas and Fahal, 91 between Khalas and Sukry, and 50 between Fahal and Sukry (Table 5). Such a distribution implies that Fahal and Sukry are more related than either one of them to Khalas.

### Repetitive Sequences

**Figure 5:**
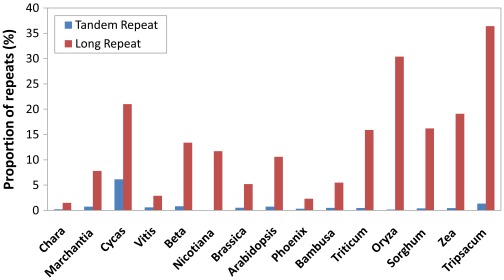
Percentage of long repeats and tandem repeats of 15 mt genomes. We analyzed long repeats (repeat unit >50 bp) using REPuter [63] and tandem repeats based on Tandem Repeat Finder [64] (see Materials and Methods for details). The genus names are used to represent the sequenced mitochondrial genomes and arranged according to their relative phylogenetic positions in Figure 2.

*P. dactylifera* mt genome has much less repetitive sequences as compared to those of other known angiosperms [44]. Only one long palindromic sequence with repeat unit longer than 1000 bp was identified (Table S9) and no inverted repeats were found. Overall, long repeats (>50 bp) only account for 2.3% of the genome, even lower than that of *Vitis* (2.9%) and *Vigna radiata* (2.7%) [45], which contain the lowest repeat contents among sequenced angiosperm mt genomes, whereas *Tripsacum* and *Oryza* contains 36.4% and 30.4% long repetitive sequences, respectively. This situation also applies to tandem repeats, which constitute only 0.33% of the genome (Table S10). Among the examined 15 plant mt genomes, whose tandem repeat contents range from 0.08% (*N. tabacum*) to 6.13% (*Cycas taitungensis*), only three mt genomes, those from *N. tabacum*, *O. sativa* and *Chara vulgaris*, contain tandem repeats lower than date palm (Figure 5). It is well known that plant mitochondria are exceptionally flexible in genome size and structure, and the accumulation of repetitive sequences often results in high sequence divergence. For instance, *Cucurbita* mt genome contains 38% of short repeats (19–621 bp in length) that make it the largest reported mt genome so far [31], whereas maize expanded its mt genome size by duplication of large sequences [46]. Therefore, it is rather unusual that date palm mitochondrial genome is both lower in tandem repeat content and rare in large duplications. It seems that larger mt genomes of angiosperms tend to have shorter repeat lengths when long repeats are compared. For instance, mt genomes of *Cucurbita* (982 kb), *Vitis* (773 kb), and *P. dactylifera* (715 kb) have their largest repeat lengths of 621 bp, 651 bp, and 1,171 bp, respectively.

### Transcriptome Analysis

**Figure 6:**
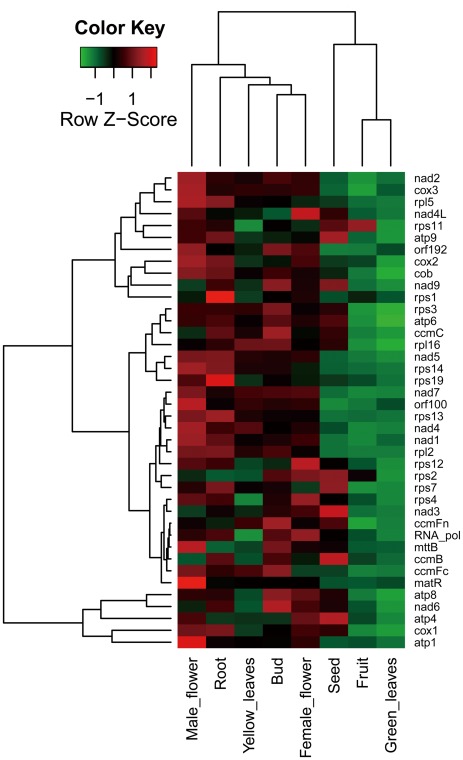
Gene expression profiles of *P. dactylifera* mitochondrion among 8 tissues. We used 40 house-keeping (conserved over diverse plant lineages) genes for hierarchical clustering (Manhattan distance method). Red and green indicate high and low levels of gene expression, respectively.

The mt-genome is transcribed by a phage-type RNA polymerase encoded in the nuclear genome [47]. The transcription process is rather complex characterized by splicing, editing, terminus processing, and multiple promoters [48]. In addition, mitochondrial genome transcription is reported to be capable of adapting to specific regulation [49]. Here, in order to better understand tissue-specific mt gene regulation and the contribution of mt genes to the development of different tissues, we performed a thorough transcriptome survey across eight date palm tissues (Figure 1 and Figure S2) using high-performance next-generation sequencers. We discovered that ∼30.8% regions of our assembly are transcribed (Table 6), slightly lower than that of the rice (∼48.5%) [50], with an average sequence coverage of ∼44× calculated based on 40 conserved house-keeping genes (Figure 6). On the one hand, our whole genome level gene expression profiling indicated that two tissues, green leaf and fruit, have the most abundant transcripts (Figure S2) but have the lowest gene expression level in terms of RPKM value (reads per kilobase of exon model per million mapped reads) [51] (Figure 6). Male and female flowers, root, and bud, on the other hand, tend to have higher gene expression levels but less in transcript abundance than the leaves. We assume that developing tissues, such as yellow leaf, bud, and root, need more energy than the relatively mature tissues, such as green leaf and fruit. By the same token, the highly expressed genes in female and male flowers are possibly related to flower development that not only depends on a set of nuclear genes but also on the coordinated action of mitochondrial genes [52]. It is possible that the variable expression of mt genome-encoded genes is relevant to the copy number variation of mt genomes (similar to the number of mitochondria per cell) [53], [54] or its changing status in tissue development. In addition, several other obvious tissue-specific gene expression patterns can be observed. First, consistent with a previous study that *atp1* gene prefers to express in pollen mother cells [55], we also detected that the transcript of *atp1* is obviously more abundant in male flower than in other tissues examined. There is another gene *matR* that encodes a maturase-related protein also expressed in a relative higher level. Previous study revealed that this gene suffers from modest RNA editing in maize and soybean and was predicted to be functional [56]. Our results here indicate that this gene should have utmost importance in male flower development. Second, the maturation process of yellow to green leaves seems to involve the suppression of about half of the 40 mt house-keeping genes, and the fact is further confirmed that developing tissues are more affected by mt gene expression. Third, during seed maturation, half of the genes were found to be up-regulated when compared to the mt gene expression pattern in fruit. Fourth, interestingly, we found that the gene expression patterns of yellow leaves and seeds are quite distinct–down-regulated genes in one tissue tend to be highly regulated in the other tissue–except that of *ccmFn*, *cox2*, *rps1*, *rps3*, and *rps19* which have no obvious differences between these two tissues. Fifth, two genes, *rps1* and *rps19,* are found clearly highly expressed in root as compared to other tissues. The functional roles they play in root development still need further experimental confirmation. Sixth, consistent with previous studies, rRNA gene transcripts are found to be more abundant, ∼9–13 fold than protein coding genes [57], but our large-scale transcriptomic analysis reveals a much higher transcription level changes ∼50–400 fold than the average level of protein-coding genes according to RPKM values, and the order of expression levels for the ribosomal RNAs is 5S rRNA >26S rRNA >18S rRNA.

**Table 6:** The transcript coverage of *P.dactylifera* mt genome.

Reads^a^	Length (bp)^b^	Percentage (%)^c^
= 0	494769	69.20
1–9	136935	19.15
10–99	69116	9.67
100–999	10734	1.50
1000–9999	3020	0.42
>10000	427	0.060

a Read number in each genome position. “ = 0" means no transcription activity was observed and the larger the number the higher the gene expression level. Average coverage of 40 highly-conserved genes (∼51,000 bp in length) in *P. dactylifera* mt genome is 44.26×.

b Total length of genomic sequences defined for transcript expression level.

c The proportion of transcribed region relative to the whole mt genome.

### Conclusion

As the first of the palm family plants, *P. dactylifera* mt genome displays several unique features. First, it positions at the root of the known monocot mt genomes. Second, it has a very low level of repeat content and shows abundant RNA editing events. Third, it exhibits a high level of chloroplast sequence insertions as compared to other known angiosperm mt genomes. Furthermore, our large-scale transcriptome analysis revealed that ∼30.8% of its sequences are transcribed and show obvious tissue-specific gene regulation patterns, among which both female and male flowers, root, and bud exhibit higher gene expressions than other sampled tissues. Our complete mt genome sequence assembly represents a new addition to the growing number of plant mt genomes in the public databases and paves a way for further investigations on mitochondrial biology of seed plants.

## Materials and Methods

### Plant Materials

We used three domestic *P. dactylifera* cultivars, Khalas (male and female), Sukry (female), and Fahal (male), for this study. Tissue samples from adult date palm trees grown in Al-Hasa Oasis of Kingdom of Saudi Arabia are harvested, including soft bud, flower (male and female), fruit, root, yellow leaf (young), and green leaf (old). We disinfected the samples with 75% ethanol and froze them in liquid nitrogen immediately. For longer term storage, they are stored in −80°C freezer until use.

### Genomic and RNA Sequencing

The *P. dactylifera* mt genome sequences are produced as part of the Date Palm Genome Project (DPGP, a joint effort between KACST and CAS). Genomic DNA was extracted from 50 g soft bud tissues according to the CTAB-based method. We used 5 µg purified DNA for shearing and constructing fragment libraries following the GS FLX Titanium general library preparation protocol. The ssDNA libraries were amplified with emulsion-PCR and enriched, and the samples were sequenced on Roche/454 GS FLX platform.

SOLiD long mate pair (LMP) libraries of the three cultivars were constructed by following SOLiD Library Preparation Guide (SOLiD 4.0) and at least 20 µg genomic DNA was used depending on different insert sizes (600–6000 bp). After emulsion PCR and beads enrichment (EZ beads system, AB), template beads of each LMP library were deposited to 2 quarter of slide and then loaded onto a SOLiD 4.0 instrument.

For transcriptomic study, tissue samples were grinded into fine powder followed by CTAB-based RNA extraction, and 2.5 M LiCl was used to remove polysaccharides. 0.5 µg rRNA-depleted total RNA (RiboMinus Plant Kit, Invitrogen) were used to construct transcriptomic libraries according to the instruction from SOLiD Total RNA-Seq Kit.

### Sequence Assembly and Validation

We separated candidate mt genome reads from eight Roche/454 GS FLX runs based on 40 published plant mt genome sequences (identity ≥80% and E-value ≤10^−5^). About 1.5 millions reads were obtained and assembled by using Newbler (version 2.3 with default parameters)–a *de novo* sequence assembly software provided by Roche. As a result, we obtained 29 mt genome contigs (total ∼438 kb) with an average length of 15 kb. These contigs were extended to 662 kb by adding additional Roche/454 reads. Subsequently, SOLiD mate-pair data (2×50 bp libraries) with insertion sizes of 1–2 kb and 3–4 kb were used to construct scaffold (50-nt overlap cutoff and less than 2-nt mismatch). A total of 3,918 homopolymers with repeat unit ranging from 5 to 11 were verified and revised based on SOLiD fragment data using BFAST program (version 0.6.4d) [58]. At last, 715,001 bp complete mt genome was assembled with an average sequence depth 130×. The final genome sequence was validated by SOLiD LMP data with insertion sizes of 3–4 kb, 4–5 kb, and 5–6 kb in a 2 kb sliding window with variable step sizes; we show the result from an analysis using 5–6 kb insert size in 15-kb step size in Figure 1.

The complete sequence of the date palm mt genome was deposited to GenBank (accession number JN375330).

### Sequence Annotation

A preliminary annotation was carried out by mapping final genome sequence with BLAST (identity >90% and overlap>90%) [59], [60] hits to known mitochondrial genes, and subsequently by testing for consistency of the ORFs using NCBI online tool the ORF finder (http://www.ncbi.nlm.nih.gov/projects/gorf/, the standard genetic code was applied). The exact gene and exon boundaries were determined by alignment of homologous genes from several common mt genomes (Table S2) and verified based on transcriptomic data. The tRNA genes were identified by using a local chloroplast and mitochondrial tRNA database, BLAST search tools [59], [60], and the help of tRNAscan-SE program (version 1.4 and default parameters were used) [61]. Both group I and group II introns were predicted by using an online software Rfam (version 10.1; http://rfam.sanger.ac.uk/; default parameters) [62]. Homology search using BLAST [59], [60] was carried out to identify chloroplast-derived regions in the mt genome assembly (over 80% sequence identity; E value ≤1e-5; length >50 bp).

### RNA Editing Analyses

We predicted putative RNA editing sites in protein-coding genes using the PREP-mt web-based program (http://prep.unl.edu/) [40]. To achieve a balanced tradeoff between the number of false positive and false negative sites, the cutoff score (C-value) was set to 0.6 as suggested by the author. All other parameters are set to default values. We also verified some of the RNA editing sites in five genes (*atp1*, *atp4*, *atp9*, *rpl116,* and *rps19*) across the two leaf tissues (yellow and green leaves) using cDNA data from Roche/454 GS FLX system (NCBI accession number SRA045434.3). The five genes are chosen randomly, whose cDNA sequences are full-length and better in quality.

### SNP Analysis

We carried out both intra-varietal and inter-varietal SNP analysis across three cultivars: Khalas, Fahal, and Sukry. Three runs of SOLiD LMP reads for each cultivar (about 60 Gb) were mapped to the reference mt genome (Khalas) by using BioScope software (version 1.3). The mapping results were then used for SNP identification based on a Bayesian algorithm according to the BioScope Software User Guide.

### Analysis of Repetitive Sequences

We identified repetitive sequences, including forward, palindromic, reverse, and complemented repeats, using the REPuter (version 2.74; with a minimal length of 50 bp and 3 mismatches) [63]. We removed overlapped repeats manually and obtained information on tandem repeats using a tandem repeat finder (http://tandem.bu.edu/trf/trf.html; default parameters were used) [64].

### Phylogenetic Analyses

We used 22 protein-coding genes (*atp1*, *atp4*, *atp6*, *atp8*, *atp9*, *ccmC*, *ccmFn*, *cob*, *cox1*, *cox2*, *cox3*, *nad1*, *nad2*, *nad3*, *nad4*, *nad4L*, *nad5*, *nad6*, *nad9*, *rps3*, *rps4,* and *rps12*) common to 15 plant mt genomes for our phylogenetic analysis. We aligned the sequences using Clustalw2 (default parameters were used) [65], removed ambiguously aligned regions based on Gblocks (version 0.91b; minimum number of sequences for a conserved position and flanking position is set to 10 and 15, respectively; no more than eight contiguous non-conserved positions and no gap are allowed) [66], and concatenated the sequences. The maximum-likelihood tree was constructed by using PHYML (version 3.0) [67] under HKY85+Γ4 model (*C. vulgaris* is used as outgroup). The bootstrap value was set to 100. All other parameters are set as default.

### Transcriptome Analyses

We used transcriptome data from bud, root, seed, fruit, male and female flowers, yellow and green leaves of cultivar Khalas. On average, ∼700,000 SOLiD reads (50 bp with 3 mismatches or less) are used from the libraries. RPKM values are measured (reads per kilobase of exon model per million mapped reads) [51] and used to estimate gene expression, which are calculated according to:
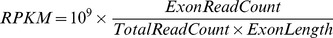



## Supporting Information

Figure S1 **GC content variations between new and old chloroplast-derived sequences.** We defined chloroplast-derived sequences unique to *P. dactylifera* mitochondrial genome as “New" and those shared by other plant mt genomes as “Old".
(TIF)

Figure S2 **Transcriptome analysis across eight tissues.** FF, female flower (∼422,000 reads); MF, male flower (∼589,000 reads); F, fruit (∼1,048,000 reads); S, seed (∼179,000 reads); B, bud (∼457,000 reads); GL: green leaf (∼2,388,000 reads); YL, yellow leaf (∼606,000 reads); R, root (∼545,000 reads); P, genes on the positive strand; N, genes on the negative stand; and CP, chloroplast-derived regions. Their RPKM values (transformed using log10) range from 0 to 9 for genes on the positive strand and 0 to −9 for genes on the negative strand.
(TIF)

Table S1 **15 plant mt genomes used in this study.**
(PDF)

Table S2 **Genes in 12 angiosperm mt genomes.**
(PDF)

Table S3 **tRNA gene content of** ***P. dactylifera*** **mt genome.**
(PDF)

Table S4 **Chloroplast-derived sequences in** ***P. dactylifera*** **mt genome.**
(PDF)

Table S5 **RNA editing sites in three different plant mt genomes.**
(PDF)

Table S6 **RNA editing validation of five genes in two tissues based on GS FLX reads.**
(PDF)

Table S7 **Inter-varietal SNPs in coding regions among the three cultivars.**
(PDF)

Table S8 **Inter-varietal SNPs in non-coding regions among the three cultivars.**
(PDF)

Table S9 **Long repeats (repeat unit >50 bp) in** ***P. dactylifera*** **mt genome.**
(PDF)

Table S10 **Tandem repeats in** ***P. dactylifera*** **mt genome.**
(PDF)
