# Supplementary figures and images for: A Complete Sequence and Transcriptomic Analyses of Date Palm (Phoenix dactylifera L.) Mitochondrial Genome
Source: PLoS One. 2012 May 24;7(5):e37164. doi: 10.1371/journal.pone.0037164 (PMC3360038; doi:10.1371/journal.pone.0037164)

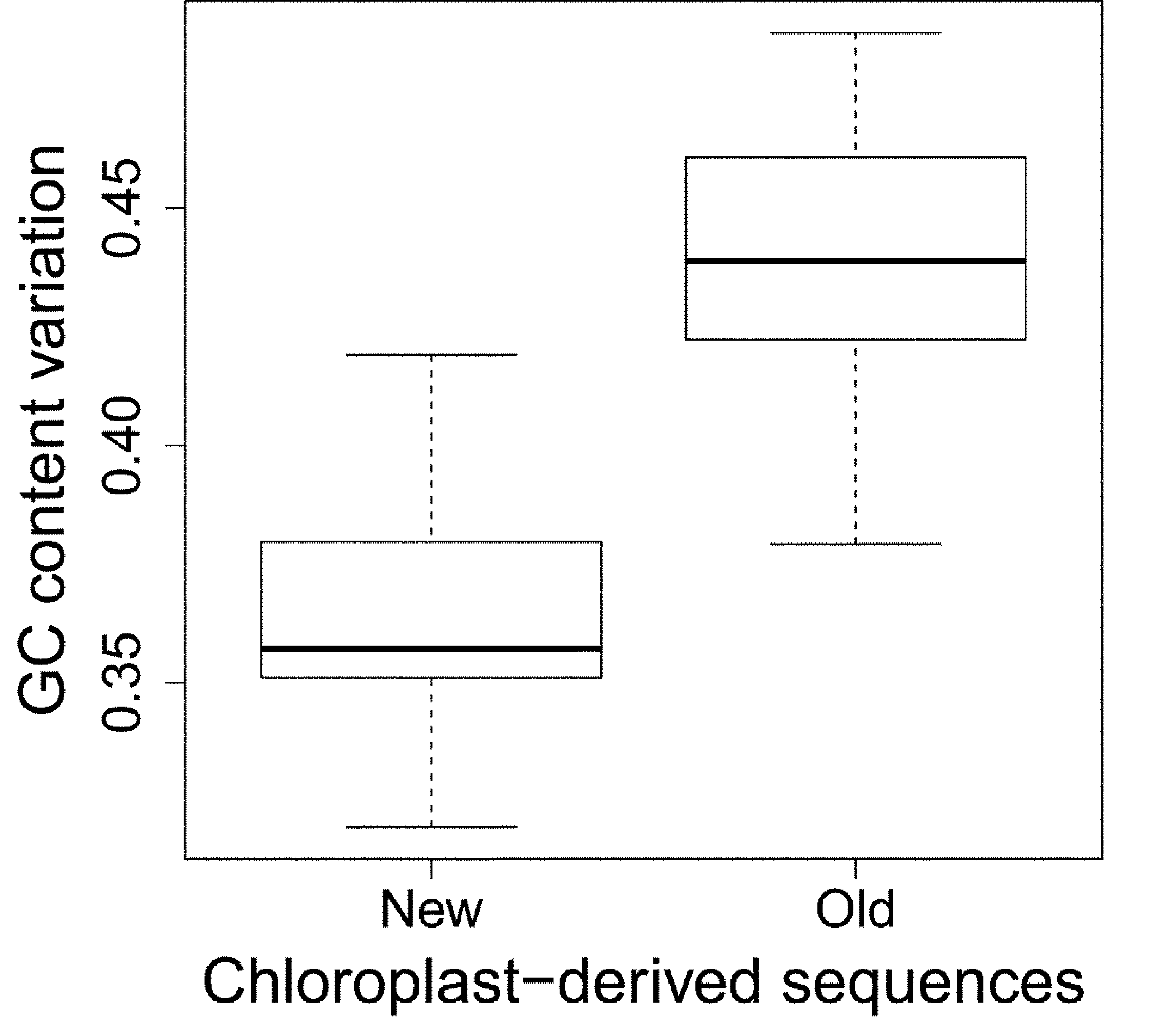

Supplement: Figure S1 — GC content variations between new and old chloroplast-derived sequences. We defined chloroplast-derived sequences unique to P. dactylifera mitochondrial genome as “New" and those shared by other plant mt genomes as “Old". (TIF) [file pone.0037164.s001.tif]

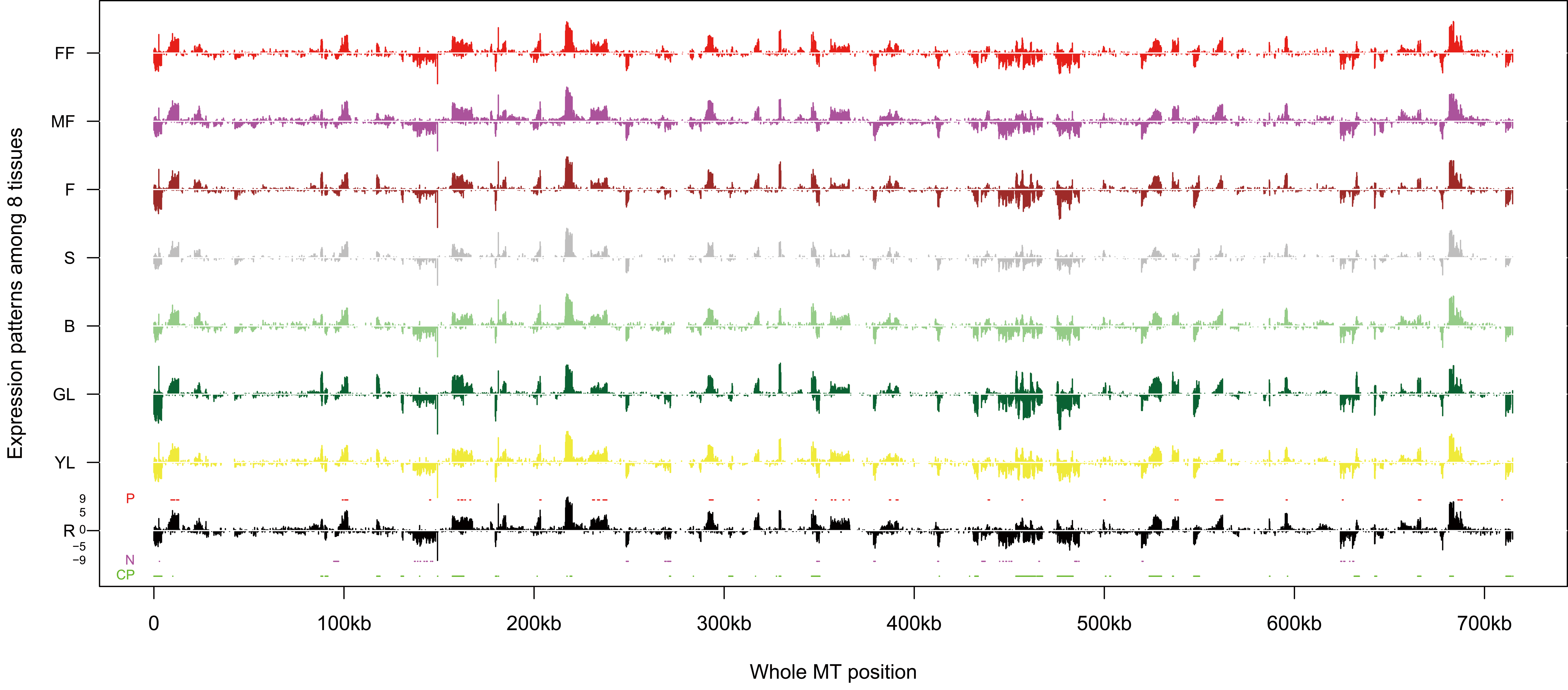

Supplement: Figure S2 — Transcriptome analysis across eight tissues. FF, female flower (∼422,000 reads); MF, male flower (∼589,000 reads); F, fruit (∼1,048,000 reads); S, seed (∼179,000 reads); B, bud (∼457,000 reads); GL: green leaf (∼2,388,000 reads); YL, yellow leaf (∼606,000 reads); R, root (∼545,000 reads); P, genes on the positive strand; N, genes on the negative stand; and CP, chloroplast-derived regions. Their RPKM values (transformed using log10) range from 0 to 9 for genes on the positive strand and 0 to −9 for genes on the negative strand. (TIF) [file pone.0037164.s002.tif]
