# Supplementary material for: A Complete Sequence and Transcriptomic Analyses of Date Palm (Phoenix dactylifera L.) Mitochondrial Genome
Source: PLoS One. 2012 May 24;7(5):e37164. doi: 10.1371/journal.pone.0037164 (PMC3360038; doi:10.1371/journal.pone.0037164)
Supplement: Table S1 — 15 plant mt genomes used in this study. (PDF) [file pone.0037164.s003.pdf]

Table S1. 15 plant mt genomes used in this study.

Classfication	Scientific Name	Common Name	Accession Number	Reference
**Algae**				
Charophyta	*Chara vulgaris*	Green algae	NC_005255	[1]
**Land plants**				
Bryophyta	*Marchantia polymorpha*	Liverwort	NC_001660	[2]
**Seed plants**				
Gymnosperm				
Cycads	*Cycas taitungensis*	Emperor sago	NC_010303	[3]
Angiosperm				
Monocots	*Phoenix dactylifera*	Date palm	NC_016740	This study
	Bambusa oldhamii	Giant timber bamboo	EU365401	NA
	Triticum aestivum	Wheat	NC_007579	[4]
	Oryza sativa	Rice	NC_011033	[5]
	Sorghum bicolor	Sorghum	NC_008360	NA
	Tripsacum dactyloides	Gama grass	NC_008362	NA
	Zea mays	Maize	NC_007982	[6]
Dicots	*Beta vulgaris*	Sugar beet	NC_002511	[7]
	Brassica napus	Oilseed rape	NC_008285	[8]
	Arabidopsis thaliana	Mouse-ear cress	NC_001284	[9]
	Nicotiana tabacum	Tobacco	NC_006581	[10]
	Vitis vinifera	Grape	NC_012119	[11]

NA: no published papers are available.
