# Supplementary material for: A Complete Sequence and Transcriptomic Analyses of Date Palm (Phoenix dactylifera L.) Mitochondrial Genome
Source: PLoS One. 2012 May 24;7(5):e37164. doi: 10.1371/journal.pone.0037164 (PMC3360038; doi:10.1371/journal.pone.0037164)
Supplement: Table S2 — Genes in 12 angiosperm mt genomes. (PDF) [file pone.0037164.s004.pdf]

Table S2. Genes in 12 angiosperm mt genomes.

	Beta	Brassica	Arabidopsis	Nicotiana	Vitis	Phoenix	Bambusa	Triticum	Oryza	Sorghum	Tripsacum	Zea
**Respiratory chain complex I**												
nad1	+	+	+	+	+	+	+	-	+	+	+	+
nad2	+	+	+	+	+	+	+	+	+	+	+	+
nad3	+	+	+	+	+	+	+	+	+	+	+	+
nad4	+	+	+	+	+	+	+	+	+	+	+	+
nad4L	+	+	+	+	+	+	+	-	+	+	+	+
nad5	+	+	+	+	+	+	+	+	+	+	+	+
nad6	+	+	+	+	+	+	+	+	+	+	+	+
nad7	+	+	+	+	+	+	+	+	+	+	+	+
nad9	+	+	+	+	+	+	+	+	+	+	+	+
**Respiratory chain complex II**												
sdh3	-	-	-	+	+	-	-	-	-	-	-	-
sdh4	-	-	-	+	+	-	-	-	-	-	-	-
**Respiratory chain complex III**												
cob	+	+	+	+	+	+	+	+	+	+	+	+
**Respiratory chain complex IV**												
cox1	+	+	+	+	+	+	+	+	+	+	+	+
cox2	+	+	+	+	+	+	+	+	+	+	+	+
cox3	+	+	+	+	+	+	+	+	+	+	+	+
**Respiratory chain complex V**												
atp1	+	+	+	+	+	+	+	+	+	+	+	+
atp4	+	+	+	+	+	+	+	+	+	+	+	+
atp6	+	+	+	+	+	+	+	+	+	+	+	+
atp8	+	+	+	+	+	+	+	+	+	+	+	+
atp9	+	+	+	+	+	+	+	+	+	+	+	+
**Cytochrome c biogenesis**												
ccmB	+	+	+	+	+	+	+	+	+	+	+	+
ccmC	-	+	+	+	+	+	+	+	+	+	+	+
ccmFC	+	+	+	+	+	+	+	+	+	+	+	+
ccmFN	+	+	+	+	+	+	+	+	+	+	+	+
**Ribosomal proteins**												
rpl2	-	+	+	+	+	+	-	-	+	-	-	-
rpl5	+	+	+	+	+	+	+	+	+	-	-	-
rpl16	-	+	+	+	+	+	+	+	+	+	+	+
rps1	-	-	-	+	+	+	+	+	+	+	+	+
rps2	-	-	-	-	-	+	-	-	+	+	+	+
rps3	+	+	+	+	+	+	+	+	+	+	+	+
rps4	+	+	+	+	+	+	+	+	+	+	+	+
rps7	+	+	+	-	+	+	+	+	+	+	+	+
rps10	-	-	-	+	+	-	-	-	-	-	-	-
rps11	-	-	-	-	-	+	-	-	-	-	-	-
rps12	+	+	+	+	+	+	+	+	+	+	+	+
rps13	+	-	-	+	+	+	+	+	+	+	+	+
rps14	-	+	-	-	+	+	-	-	-	-	-	-
rps19	-	-	-	+	+	+	-	-	+	-	-	-

**Other proteins**												
RNA_pol	-	-	-	-	+	+	-	-	-	-	-	-
tatC	+	+	+	+	-	-	+	+	+	+	+	+
mttB	+	+	+	+	-	+	+	+	+	+	+	+
matr	+	+	+	+	+	+	+	+	+	+	+	+

The genus names are used to represent the sequenced mitochondrial genomes.
Presence (+) and absence (-) of proteins are from references [6,8,9,12,13,14,15].
