# Supplementary material for: A Complete Sequence and Transcriptomic Analyses of Date Palm (Phoenix dactylifera L.) Mitochondrial Genome
Source: PLoS One. 2012 May 24;7(5):e37164. doi: 10.1371/journal.pone.0037164 (PMC3360038; doi:10.1371/journal.pone.0037164)
Supplement: Table S3 — tRNA gene content of P. dactylifera mt genome. (PDF) [file pone.0037164.s005.pdf]

Table S3. tRNA gene content of *P. dactylifera* mt genome.

Type	Strand	Intron Begin	Intron End	Length(bp)
trnC-GCA	+	-	-	73
trnC-GCA[a]	+	-	-	71
trnD-GUC	+	-	-	74
trnE-UUC	-	-	-	72
trnF-GAA	-	-	-	73
trnF-GAA[a]	-	-	-	74
trnG-GCC[a]	+	-	-	71
trnH-GUG[a]	-	-	-	74
trnI-CAU	-	-	-	74
trnI-CAU	-	-	-	74
trnK-UUU	+	-	-	73
trnK-UUU	-	211181	211177	72
trnfM-CAU	+	-	-	74
trnfM-CAU	-	-	-	74
trnM-CAU[a]	-	-	-	73
trnN-AUU	+	643129	643231	187
trnN-GUU[a]	-	-	-	72
trnP-GGG[a]	-	-	-	76
trnP-UGG	+	-	-	66
trnP-UGG	-	-	-	75
trnQ-UUG	-	-	-	72
trnS-GCU	-	-	-	88
trnS-GGA[a]	+	-	-	87
trnS-UGA	+	-	-	87
trnS-UGA[a]	-	-	-	93
trnSup-CUA	-	643292	643122	247
trnT-UGU[a]	-	-	-	73
trnW-CCA[a]	-	-	-	74
trnW-CCA[a]	-	-	-	74
trnY-GUA	-	-	-	83

[a]Chloroplast-derived tRNA genes.
