# Supplementary material for: A Complete Sequence and Transcriptomic Analyses of Date Palm (Phoenix dactylifera L.) Mitochondrial Genome
Source: PLoS One. 2012 May 24;7(5):e37164. doi: 10.1371/journal.pone.0037164 (PMC3360038; doi:10.1371/journal.pone.0037164)
Supplement: Table S4 — Chloroplast-derived sequences in P. dactylifera mt genome. (PDF) [file pone.0037164.s006.pdf]

Table S4. Chloroplast-derived sequences in *P. dactylifera* mt genome.

Length	Start	End	Gene	Length	Start	End	Gene
6521	523589	530109	*rpoB*/***rpoC1***	768	642268	643035	-
3811	475991	479801	***matK*/*psbA*/***trnK-UUU*	683	481655	482337	-
3792	457440	461231	***psaB*/*rps14*/***trnfM-CAU*	668	431878	432545	*atpF*
3239	159412	162650	***cemA*/*petA*/***psaI*/***ycf4***	625	475246	475870	*rps19*/***trnH-GUC***
3092	547003	550094	***trnS-GGA*/*trnT-UGU***	577	461232	461808	*petD*
2821	464513	467333	-	572	432720	433291	-
2566	346519	349084	***rpl20*/*rps18***	506	349651	350156	*psaJ*
2492	1732	4223	*psbB*/***psbH*/*psbN*/*psbT***	494	535882	536375	-
2109	302457	304565	***trnC-GCA***	451	271397	271847	***rpl14***
1990	712054	714043	***atpI*/*rps2***	421	631748	632168	*ndhK*
1952	157001	158952	***accD***	406	349167	349572	***rpl33***
1940	461866	463805	***rpl36*/*rpoA*/***rps11*	383	346101	346483	-
1720	117234	118953	*ndhA*/***ndhI***	360	483235	483594	*rpl16*
1694	1	1694	***petB***	287	633869	634155	*trnL-UAA*
1321	454531	455851	***psbE*/*psbF*/*psbJ*/*psbL***	283	711284	711566	*atpH*
1285	130051	131335	*atpA*	281	633482	633762	***trnF-GAA***
1028	632210	633237	***ndhJ***	270	586885	587154	-
1018	456443	457460	***petG*/*petL*/*trnW-CCA***	267	500598	500864	-
967	87871	88837	*atpB*	195	350166	350360	*trnP-UGG*
927	90092	91018	-	194	91361	91554	***trnP-UGG***
899	328935	329833	*rbcL*	156	479939	480094	*trnK-UUU*
880	664814	665693	***rpl14*/***rpl16*	151	454342	454492	***trnG-GCC***
862	453450	454311	***psbZ*/*trnS-UGA***	124	139760	139883	*trnM-CAU*
823	179701	180523	*trnA-UGC*/*trnI-GAU*	109	91158	91266	***trnW-CCA***
784	665782	666565	***infA*/***rps8*	83	201609	201691	***trnN-GUU***
778	480826	481603	*rps16*	58	413150	413207	-

Chloroplast-derived sequences longer than 50bp were displayed.

Genes with partial region (from 50% to 95%) shown in regular font and genes with complete sequence (> 95%) are highlighted in bold; different genes indentified in the same region are separated by an oblique line.

-, no genes are identified.
