# Supplementary material for: A Complete Sequence and Transcriptomic Analyses of Date Palm (Phoenix dactylifera L.) Mitochondrial Genome
Source: PLoS One. 2012 May 24;7(5):e37164. doi: 10.1371/journal.pone.0037164 (PMC3360038; doi:10.1371/journal.pone.0037164)
Supplement: Table S5 — RNA editing sites in three different plant mt genomes. (PDF) [file pone.0037164.s007.pdf]

Table S5. RNA editing sites in three different plant mt genomes.

Gene	Phoenix	Arabidopsis	Oryza	Shared
atp1	11	5	5	2
atp4	8	8	9	4
atp6	26	1	16	0
atp8	4	0	4	0
atp9	10	4	8	2
ccmB	35	39	35	21
ccmC	33	28	35	21
ccmFc	11	16	27	5
ccmFn	37	22	31	10
cob	20	7	19	6
cox1	27	0	4	0
cox2	17	15	19	7
cox3	16	8	1	0
matR	16	9	0	0
mttB	24	24	33	13
nad1	27	24	23	12
nad2	35	29	30	11
nad3	20	12	15	8
nad4	59	32	20	12
nad4L	11	9	10	5
nad5	32	27	11	7
nad6	17	10	18	6
nad7	35	27	32	0
nad9	13	7	12	6
rpl2	4	1	1	0
rpl5	10	10	1	1
rps12	7	8	0	0
rps3	12	13	10	3
rps4	13	15	15	7
rps7	2	0	2	0
**Total**	**592**	**410**	**446**	**169**

The genus names are used to represent the sequenced mitochondrial genomes.
