# Supplementary material for: A Complete Sequence and Transcriptomic Analyses of Date Palm (Phoenix dactylifera L.) Mitochondrial Genome
Source: PLoS One. 2012 May 24;7(5):e37164. doi: 10.1371/journal.pone.0037164 (PMC3360038; doi:10.1371/journal.pone.0037164)
Supplement: Table S6 — RNA editing validation of five genes in two tissues based on GS FLX reads. (PDF) [file pone.0037164.s008.pdf]

Table S6. RNA editing validation of five genes in two tissues based on GS FLX reads.

	Prediction	Green leaves					Yellow leaves				
Gene	Position	Position	Ref	Edit	Reads	%	Position	Ref	Edit	Reads	%
atp1	-	246	C	T	6	67	-	-	-	-	-
	670	√	C	T	13	100	√	C	T	9	100
	971	√	C	T	29	100	√	C	T	17	100
	1039	√	C	T	34	100	√	C	T	16	100
	1064	√	C	T	35	100	√	C	T	15	93
	1168	√	C	T	30	100	√	C	T	14	100
	1178	√	C	T	30	90	√	C	T	13	92
	1262	√	C	T	35	91	√	C	T	18	89
	1292	√	C	T	27	96	√	C	T	15	87
	1415	√	C	T	30	97	√	C	T	14	93
	-	1431	C	T	29	79	1431	C	T	14	50
	1490	√	C	T	21	100	√	C	T	9	100
	1499	√	C	T	19	89	√	C	T	9	100
atp4	56	√	C	T	4	100	√	C	T	6	100
	-	59	C	T	4	100	59	C	T	6	100
	71	√	C	T	4	100	√	C	T	6	100
	89	√	C	T	4	100	√	C	T	6	100
	118	√	C	T	4	100	√	C	T	6	100
	215	√	C	T	9	100	√	C	T	10	100
	-	227	C	T	9	100	227	C	T	10	100
	248	√	C	T	9	100	√	C	T	10	80
	395	√	C	T	8	75	√	C	T	8	87
	416	√	C	T	8	100	√	C	T	7	100
atp9	20	√	C	T	88	98	√	C	T	88	93
	50	√	C	T	101	94	√	C	T	96	90
	82	√	C	T	121	45	√	C	T	107	95
	92	√	C	T	121	95	√	C	T	105	89
	134	√	C	T	115	98	√	C	T	96	90
	182	√	C	T	110	97	√	C	T	95	97
	191	√	C	T	107	94	√	C	T	93	92
	203	√	C	T	105	96	√	C	T	91	92
	212	√	C	T	105	98	√	C	T	91	89
	223	√	C	T	99	98	√	C	T	83	92
rpl16	-	-	-	-	-	-	15	C	T	9	100
	-	48	C	T	3	100	48	C	T	13	100
	79	√	C	T	3	100	√	C	T	13	92
	98	√	C	T	3	100	√	C	T	15	87
	184	√	C	T	5	100	√	C	T	17	100
	-	225	C	T	5	100	225	C	T	17	82
	227	√	C	T	5	100	√	C	T	17	100
	287	√	C	T	5	100	√	C	T	18	94
	355	√	C	T	5	100	√	C	T	19	95
	-	444	C	T	15	100	444	C	T	13	100
	458	√	C	T	15	100	√	C	T	12	100
	524	√	C	T	13	100	√	C	T	12	100
	530	√	C	T	13	100	√	C	T	12	100
rps19	116	√	C	T	20	95	√	C	T	23	91
	-	138	C	T	20	50	138	C	T	24	54
	163	√	C	T	17	94	√	C	T	21	95
	164	-	-	-	-	-	-	-	-	-	-
	-	221	C	T	16	100	221	C	T	22	91

The RNA editing sites are confirmed based on Roche/454 cDNA sequences.
-, no editing found.
√, editing found in the same position.
