# Supplementary material for: A Complete Sequence and Transcriptomic Analyses of Date Palm (Phoenix dactylifera L.) Mitochondrial Genome
Source: PLoS One. 2012 May 24;7(5):e37164. doi: 10.1371/journal.pone.0037164 (PMC3360038; doi:10.1371/journal.pone.0037164)
Supplement: Table S7 — Inter-varietal SNPs in coding regions among the three cultivars. (PDF) [file pone.0037164.s009.pdf]

Table S7. Inter-varietal SNPs in coding regions among the three cultivars.

Site	SNP	Khalas/Fahal/Sukry			Gene
682605	G/T/T	59.98%(1037)[a]	56.43%(381)	64.28%(1621)	18s rRNA
682760	C/C/G	66.36%(3936)	58.09%(1403)	59.48%(4812)	18s rRNA
683453	C/C/T	59.88%(1498)	59.57%(460)	69.88%(1932)	18s rRNA
683598	A/G/A	54.66%(2986)	56.14%(1181)	69.70%(3729)	18s rRNA
219188	T/T/G	73.07%(3023)	63.99%(761)	55.29%(2554)	26s rRNA
219833	T/T/C	68.74%(2086)	60.55%(1019)	50.11%(2692)	26s rRNA
219840	T/T/C	55.54%(2346)	55.11%(1134)	63.01%(2979)	26s rRNA

[a]Major genotype in percentage and number in parenthesis indicates read coverage of that base site.
