# Supplementary material for: A Complete Sequence and Transcriptomic Analyses of Date Palm (Phoenix dactylifera L.) Mitochondrial Genome
Source: PLoS One. 2012 May 24;7(5):e37164. doi: 10.1371/journal.pone.0037164 (PMC3360038; doi:10.1371/journal.pone.0037164)
Supplement: Table S8 — Inter-varietal SNPs in non-coding regions among the three cultivars. (PDF) [file pone.0037164.s010.pdf]

Table S8. Inter-varietal SNPs in non-coding regions among the three cultivars.

Site	SNP	Khalas/Fahal/Sukry		
130104	G/A/A	64.59%(4436) [a]	51.99%(1637)	52.42%(3018)
130123	T/G/G	58.09%(4784)	58.14%(1646)	55.51%(3311)
130138	A/G/G	56.83%(5091)	59.74%(1560)	55.41%(2994)
130185	T/C/C	52.69%(5456)	64.48%(2024)	66.26%(3610)
130194	A/C/C	53.45%(5297)	59.69%(1868)	62.29%(3386)
130290	A/A/G	51.40%(4706)	53.45%(1420)	64.79%(2491)
130471	T/C/C	50.02%(2205)	62.51%(891)	50.71%(1053)
130610	G/T/G	62.84%(2764)	54.71%(1773)	56.69%(1494)
130836	G/G/T	56.34%(4347)	56.21%(1772)	53.24%(2866)
130839	G/G/A	55.42%(4219)	54.47%(1722)	53.71%(2793)
131268	T/C/C	50.52%(4016)	56.67%(1454)	53.54%(1963)
131283	G/A/A	53.22%(4083)	61.54%(1820)	66.30%(2561)
179700	C/C/A	66.84%(2343)	65.85%(1025)	59.17%(2716)
179965	A/C/C	60.66%(2260)	54.30%(1070)	70.96%(2400)
179967	T/T/C	65.57%(2045)	51.80%(998)	63.56%(2212)
180069	C/C/N	51.11%(2473)	53.79%(673)	56.40%(1711)
180141	C/T/C	66.80%(3274)	52.54%(1420)	68.02%(3136)
180223	C/C/G	60.32%(2180)	77.63%(1180)	51.49%(2476)
180455	T/C/T	80.68%(2427)	53.66%(1297)	61.51%(1577)
180462	A/G/G	61.01%(2888)	59.66%(1336)	58.83%(1926)
302636	C/C/A	64.23%(2217)	52.48%(909)	56.29%(993)
302786	A/A/C	54.45%(7012)	59.33%(2186)	50.72%(4073)
302989	G/T/T	52.04%(3180)	60.57%(1395)	65.47%(1390)
303071	T/A/A	55.42%(4924)	65.93%(2090)	71.40%(2566)
303118	C/A/A	64.05%(3302)	64.68%(1308)	55.86%(1033)
303390	T/T/G	54.65%(3045)	57.59%(962)	59.05%(2127)
303545	C/A/C	100%(3755)	50.74%(1417)	100%(1583)
303744	G/T/T	69.76%(2844)	53.09%(1309)	55.04%(1468)
304135	A/C/C	66.40%(3152)	50.77%(1298)	51.29%(969)
328988	G/T/G	72.10%(2907)	51.38%(975)	55.01%(1847)
328999	G/T/T	53.48%(3654)	55.37%(1452)	54.10%(2547)
329219	A/G/G	51.61%(4811)	51.57%(1842)	71.46%(4100)
329649	T/G/G	53.03%(3485)	66.86%(1919)	58.05%(2596)
346007	C/T/T	55.33%(3656)	64.97%(1556)	64.98%(2096)
346020	G/T/T	58.79%(3116)	53.34%(1318)	53.60%(1554)
347238	T/T/A	83.46%(1953)	59.56%(586)	51.47%(851)
347241	A/A/C	79.13%(2592)	57.64%(713)	51.68%(1486)
347672	T/T/N	80.83%(8626)	77.52%(4218)	79.93%(6288)
348528	T/T/N	70.99%(4967)	69.83%(1959)	77.55%(2040)
349869	T/G/G	63.15%(2646)	71.47%(1276)	70.17%(1361)
350109	T/T/G	58.86%(2569)	52.35%(1062)	57.41%(1578)
350115	A/A/C	67.17%(2824)	58.88%(1036)	52.25%(1378)
350160	A/A/T	72.23%(2204)	50.84%(1481)	55.66%(1396)
431867	C/A/A	56.91%(5475)	65.40%(2049)	72.67%(3875)
431873	C/A/A	58.69%(5461)	66.50%(1824)	71.80%(3834)
431896	T/C/C	52.02%(4815)	64.84%(1584)	68.52%(3094)
433326	T/T/G	50.97%(2770)	60.39%(775)	51.09%(685)
433701	G/T/T	58.45%(2676)	64.24%(1138)	53.67%(1105)
433707	C/A/C	65.42%(2429)	59.14%(1028)	55.79%(846)
454198	G/T/T	65.05%(3099)	54.50%(1490)	52.93%(820)
454206	T/C/C	55.01%(3932)	54.63%(1726)	60.85%(1009)
454334	A/C/C	57.06%(1395)	58.66%(1195)	77.44%(1064)
455564	C/T/T	61.76%(3787)	56.74%(1165)	64.72%(1743)
455855	A/T/A	61.63%(503)	54.77%(283)	75.29%(170)
456181	G/T/T	65.06%(2038)	57.25%(896)	67.18%(1109)
456197	C/T/T	50.31%(3182)	59.78%(1161)	76.80%(1517)
456430	G/T/T	55.12%(1593)	61.36%(295)	59.37%(507)
456436	C/A/A	52.67%(1817)	62.86%(447)	69.47%(750)
456615	G/C/C	50.31%(5136)	61.99%(2628)	54.39%(2721)

456814	A/A/T	61.77%(1444)	58.95%(760)	52.50%(821)
461888	C/A/A	69.60%(3832)	51.25%(839)	57.84%(1632)
461894	C/C/A	73.12%(4133)	51.63%(827)	50.65%(1777)
461901	C/A/A	56.73%(5794)	65.33%(1298)	72.92%(3567)
461926	G/T/T	63.81%(5717)	64.46%(1435)	69.12%(3138)
461934	T/C/C	76.99%(4833)	56.99%(1151)	52.52%(2020)
462658	G/G/T	56.66%(5549)	53.45%(1667)	56.01%(2730)
462970	T/C/C	58.15%(6017)	50.23%(1955)	56.01%(3403)
462975	C/C/A	57.86%(5600)	50.46%(1956)	52.92%(3324)
462982	A/C/C	52.05%(6455)	55.80%(2319)	63.05%(4390)
463720	C/A/A	52.52%(2102)	76.86%(1033)	77.83%(821)
463745	A/G/G	60.19%(2615)	64.24%(727)	58.78%(1407)
463754	C/A/A	69.13%(1788)	64.37%(581)	54.72%(1155)
475901	T/G/T	100%(120)	59.49%(79)	77.32%(97)
476015	A/G/A	51.42%(3553)	54.33%(1202)	64.32%(2060)
476082	T/A/A	53.66%(5117)	65.67%(2986)	64.92%(4769)
477191	T/G/G	53.71%(3070)	54.24%(1475)	65.11%(1201)
478667	T/A/N	59.10%(2687)	50.99%(755)	59.51%(773)
478963	C/A/A	51.44%(5688)	76.14%(1626)	74.19%(2949)
478975	C/A/A	58.57%(4067)	61.51%(1156)	61.36%(1602)
478997	G/A/A	61.25%(3533)	52.64%(1233)	53.00%(1232)
479756	A/C/A	75.70%(930)	61.92%(323)	58.42%(380)
479759	G/T/G	100%(1107)	58.57%(321)	63.86%(368)
479924	A/A/T	100%(602)	67.23%(235)	53.82%(249)
481139	C/A/C	51.60%(4273)	52.19%(1481)	62.17%(1681)
481556	C/T/T	52.12%(3509)	54.48%(1173)	70.61%(1354)
481607	G/T/T	50.97%(1085)	52.77%(902)	50.20%(255)
500621	G/A/G	58.28%(5050)	54.31%(1392)	62.23%(3741)
500665	T/A/T	58.46%(3134)	54.72%(1462)	62.60%(3120)
500669	T/A/T	55.41%(3151)	56.91%(1497)	58.11%(2996)
523821	G/T/G	76.60%(4696)	60.90%(2087)	66.96%(2128)
523842	G/C/G	77.61%(4497)	50.45%(1338)	51.45%(2037)
523846	T/C/C	71.46%(4713)	53.37%(1482)	53.45%(2348)
524061	T/T/A	58.53%(5773)	50.16%(2464)	67.98%(4750)
524064	T/T/G	58.06%(5174)	51.25%(2394)	67.47%(4460)
524198	A/G/G	71.53%(3463)	53.97%(1877)	58.96%(2773)
524201	A/G/G	74.68%(3167)	50.28%(1635)	55.88%(2532)
524217	T/T/C	71.58%(3631)	50.76%(1966)	50.68%(2887)
524317	A/T/T	71.48%(4285)	57.32%(2069)	52.85%(2545)
524544	A/A/C	100%(2540)	60.27%(1329)	55.03%(1074)
524556	C/C/A	68.95%(3388)	56.66%(1735)	66.42%(1772)
529680	C/A/C	59.87%(2696)	61.77%(1823)	61.31%(1331)
535955	A/G/G	50.32%(4545)	58.71%(1073)	72.06%(2785)
550142	G/G/T	58.75%(2727)	51.78%(1010)	61.47%(1591)
631799	C/T/T	57.02%(4579)	51.09%(1658)	68.23%(2820)
631904	G/A/G	58.34%(2746)	56.85%(1372)	60.24%(1333)
633350	T/T/C	61.40%(2635)	61.05%(783)	60.76%(1073)
633771	C/C/T	60.52%(3260)	54.79%(1482)	56.29%(2018)
664930	G/A/A	58.41%(3825)	70.04%(1759)	68.85%(2931)
665242	A/T/T	58.83%(2475)	52.96%(1029)	66.42%(1358)
665617	A/A/C	56.94%(3407)	61.76%(1224)	62.10%(1409)
666534	C/T/T	50.70%(3730)	68.70%(1457)	67.28%(3044)
666550	G/T/G	75.47%(3473)	52.41%(1120)	50.49%(2238)
714937	A/A/G	57.59%(3777)	64.35%(1882)	72.09%(1677)

[a]Major genotype in percentage and number in parenthesis indicates read coverage of that base site.
