# Supplementary material for: A Complete Sequence and Transcriptomic Analyses of Date Palm (Phoenix dactylifera L.) Mitochondrial Genome
Source: PLoS One. 2012 May 24;7(5):e37164. doi: 10.1371/journal.pone.0037164 (PMC3360038; doi:10.1371/journal.pone.0037164)
Supplement: Table S9 — Long repeats (repeat unit >50 bp) in P. dactylifera mt genome. (PDF) [file pone.0037164.s011.pdf]

Table S9. Long repeats (repeat unit > 50 bp) in *P. dactylifera* mt genome.

Length	Type	Copy 1	Copy 2	Copy 3
1171	P	121590	281815	
362	P	271485	665254	
313	F	293877	685628	
204	F	167271	360506	
193	F	449744	697953	
182	P	498106	687653	
174	F	144064	628000	
169	F	413062	631365	
162	F	144238	628171	
147	P	544560	708459	
138	F	449799	698008	
122	P	412933	594984	
118	F	9644	25502	
112	F	181235	681634	
110	F	550150	643333	
109	P	544863	708204	
103	P	139640	685610	293877
100	F	246256	640028	
99	P	185520	472466	
98	P	139645	685610	293877
96	P	178294	592358	
96	P	271268	665848	
91	P	9849	139996	
91	F	164124	445426	
89	F	91167	457187	
89	F	623161	629364	
87	F	42666	444557	
86	F	165735	676967	
85	P	91439	350264	
85	P	412970	594984	
79	F	294181	685933	
79	F	483438	664829	
78	F	388449	639484	
76	P	21731	97696	
74	F	49154	695837	
74	F	388535	639570	
73	F	231145	231587	
72	P	35266	230684	
69	F	483525	664910	
68	F	455851	455878	
67	P	463738	666498	
67	P	529918	596397	
64	F	22481	698779	
64	F	235266	366118	
64	P	648415	669521	
62	P	234961	486027	
61	F	31850	254524	
61	F	185241	293822	
61	F	467346	467370	
60	F	22547	698846	
60	F	24505	602787	
60	F	202628	623167	629370
59	F	49056	695479	
57	P	216665	448694	
57	F	412975	631306	
57	F	413224	631526	
57	P	595007	631306	
57	P	648331	669611	
56	F	91360	457398	

56	P	177424	326261	
56	P	232329	360691	
56	F	388641	639672	
56	P	544761	708350	
55	P	139573	294016	685767
55	P	261015	335604	
55	F	412969	631300	
55	P	488751	561177	
54	F	167491	220228	
54	P	201582	350368	
53	F	43403	244782	
53	P	149308	681697	
53	F	218630	682970	
53	P	469891	545295	
52	P	529998	596340	
52	P	560989	698165	
51	F	234077	316650	
50	F	43294	244673	
50	F	43537	244916	
50	P	149311	181298	

Long repeats were identified using REPuter [16].
P indicates palindromic repeats.
F indicates forward repeats.
