# Supplementary material for: A Complete Sequence and Transcriptomic Analyses of Date Palm (Phoenix dactylifera L.) Mitochondrial Genome
Source: PLoS One. 2012 May 24;7(5):e37164. doi: 10.1371/journal.pone.0037164 (PMC3360038; doi:10.1371/journal.pone.0037164)
Supplement: Table S10 — Tandem repeats in P. dactylifera mt genome. (PDF) [file pone.0037164.s012.pdf]

Table S10. Tandem repeats in *P. dactylifera* mt genome.

Position	Repeat unit	Copy Number
692936--692997	31	2
119556--119629	30	3
643096--643163	27	3
455852--455946	27	4
229827--229901	26	3
463328--463376	24	2
467351--467427	24	3
542819--542865	23	2
327909--327952	22	2
327562--327603	21	2
453565--453607	21	2
692251--692290	21	2
670--715	20	2
281234--281272	19	2
643083--643164	19	4
37686--37723	19	2
197457--197528	19	4
55702--55761	18	3
339044--339076	16	2
403573--403619	18	3
467344--467434	15	6
633803--633840	17	2
55702--55734	16	2
55706--55761	16	3
120743--120775	16	2
268539--268569	16	2
533483--533527	15	3
548779--548810	16	2
616303--616337	16	2
55737--55797	16	4
123494--123536	15	3
360465--360499	15	2
655234--655263	15	2
349131--349163	14	2
55763--55800	13	3
457980--458005	13	2
109453--109477	12	2
440993--441041	12	4
453367--453396	12	3
454510--454536	12	2
479897--479922	12	2
713330--713354	12	2
453366--453406	10	4
197516--197579	10	6
467348--467422	9	9
643116--643163	8	6
455852--455946	7	14
360465--360499	5	7
403545--403580	3	12

Tandem repeats were analyzed based on Tandem Repeat Finder [17] under its default parameter settings.
